# Human In Situ Study of the effect of Bis(2-Methacryloyloxyethyl) Dimethylammonium Bromide Immobilized in Dental Composite on Controlling Mature Cariogenic Biofilm

**DOI:** 10.3390/ijms19113443

**Published:** 2018-11-02

**Authors:** Mary Anne S. Melo, Michael D. Weir, Vanara F. Passos, Juliana P. M. Rolim, Christopher D. Lynch, Lidiany K. A. Rodrigues, Hockin H. K. Xu

**Affiliations:** 1 Department of Advanced Oral Sciences and Therapeutics, University of Maryland School of Dentistry, Baltimore, MD 21201, USA; Mweir@umaryland.edu; 2 Postgraduate Program in Dentistry, Faculty of Pharmacy, Dentistry and Nursing, Federal University of Ceara, Fortaleza, CE 60430-355, Brazil; vanarapassos@hotlmail.com; 3 Faculty of Dentistry UniChristus, Fortaleza, CE 60160-230, Brazil; julianapml@unichristus.br; 4 Restorative Dentistry, University Dental School and Hospital, University College Cork, Wilton T12 K8AF, Ireland; chris.lynch@ucc.ie; 5 Center for Stem Cell Biology & Regenerative Medicine, University of Maryland School of Medicine, Baltimore, MD 21201, USA; 6 Marlene and Stewart Greenebaum Cancer Center, University of Maryland School of Medicine, Baltimore, MD 21201, USA

**Keywords:** antibacterial, biofilm, caries, dental composite, quaternary ammonium monomers, human in situ study

## Abstract

Cariogenic oral biofilms cause recurrent dental caries around composite restorations, resulting in unprosperous oral health and expensive restorative treatment. Quaternary ammonium monomers that can be copolymerized with dental resin systems have been explored for the modulation of dental plaque biofilm growth over dental composite surfaces. Here, for the first time, we investigated the effect of bis(2-methacryloyloxyethyl) dimethylammonium bromide (QADM) on human overlying mature oral biofilms grown intra-orally in human participants for 7–14 days. Seventeen volunteers wore palatal devices containing composite specimens containing 10% by mass of QADM or a control composite without QADM. After 7 and 14 days, the adherent biofilms were collected to determine bacterial counts via colony-forming unit (CFU) counts. Biofilm viability, chronological changes, and percentage coverage were also determined through live/dead staining. QADM composites caused a significant inhibition of *Streptococcus mutans* biofilm formation for up to seven days. No difference in the CFU values were found for the 14-day period. Our findings suggest that: (1) QADM composites were successful in inhibiting 1–3-day biofilms in the oral environment in vivo; (2) QADM significantly reduced the portion of the *S. mutans* group; and (3) stronger antibiofilm activity is required for the control of mature long-term cariogenic biofilms. Contact-killing strategies using dental materials aimed at preventing or at least reducing high numbers of cariogenic bacteria seem to be a promising approach in patients at high risk of the recurrence of dental caries around composites.

## 1. Introduction

In the past decade, dental materials research has intensified attempts at reducing or modulating dental plaque biofilm growth over dental composite surfaces [1] because recurrent caries around restorations (CARS) are identified as some of the major reasons for the failure of composite restorations [2,3]. Replacement rates of failed restorations have been reported to be 37–70%, with consequences that can seriously compromise oral health status [4,5]. CARS are frequently located at the gingival margins of the proximal restorations, which are common areas for food impaction [6]. Patients consider the cleaning of dental biofilm at the proximal space challenging, and very often fail to control biofilm build-up over time.

Dental caries are marked by continuous mineral loss promoted by organic acids released by bacteria after sugar metabolization [7], mainly streptococci from the *mutans* group. Cariogenic bacteria are characterized as pathologically shifted species, with the ability to generate large amounts of acid and to survive in acidic microenvironments [8]. The introduction of novel treatment approaches, supplementary to conventional therapeutic strategies, is thus considered as crucial for the efficient control of CARS. Cariogenic oral biofilms influence the initiation and progression of carious lesions not just in their primary development but also in their recurrence [9]. Reports in the literature have stated a temporal relationship between changes in biofilm composition and enamel demineralization following exposure to sucrose [10,11]. An undisturbed dental biofilm exposed to frequent sucrose leads to enamel demineralization after seven days of biofilm accumulation [10]. As the cariogenic biofilm becomes more mature, some acidogenic and aciduric bacteria become dominant in the biofilm [12].

Resin composites facilitate cariogenic biofilm growth [13]. Dental monomers such as bisphenol A-glycidyl dimethacrylate (BisGMA) and triethylene glycol dimethacrylate (TEGMA) may alter the metabolism and promote the proliferation of *Streptococcus mutans* [14]. Therefore, the synthesis of free radical monomers that have quaternary ammonium groups in their chemical structures paved the way for a noninvasive, biofilm-targeted method that can be used against oral biofilms [15]. Reactive and easily miscible quaternary ammonium monomers have the advantage of copolymerizing with the current dental resin systems through covalent bonding with the polymer network. These polymers are referred to as nonleaching antimicrobial or contact-killing agents. The antibacterial action results from the direct contact of the polymer with the microorganisms, with no release of active molecules. Although the exact antimicrobial mechanism of action has not been fully elucidated, the predominant mode of action is disruption of the cell membrane [16]. This imparts a durable and permanent antibacterial capability to dental composites.

Studies have presented different positions of the functional groups and alkyl chain length for improved balance between mechanical properties, antibacterial effects, and biocompatibility [17]. The majority of the synthetic quaternary ammonium monomers have only one methacrylate group, as monomethacrylates. Incorporating a high content of monomethacrylates could compromise the overall cross-linked polymer matrix [18].

Several in vitro studies have investigated the antibacterial performance of bis(2-methacryloyloxyethyl) dimethylammonium bromide (QADM), a quaternary ammonium monomer containing two methacrylate groups [18]. QADM was loaded at 10 wt % into different parental formulations, such as commercial and experimental adhesive systems [19,20] and nanocomposites [21], rendering reductions in *S. mutans* and total micro-organisms. Overall, these studies achieved a significant reduction of biofilm viability, metabolic activity, lactic acid, and bacterial counts using a 48-h human saliva microcosm biofilm model [22]. The incorporation of QADM also did not compromise the mechanical or bonding performance of the parental materials, and its antibacterial and mechanical properties were long-term and maintained after a one-year follow-up [23].

Although encouraging results were found in vitro [19,20,21,22], only a few studies have used native in situ dental plaque to study the effects of quaternary ammonium methacrylate [24,25]. In these studies, bacterial colonization over a short period (from hours to three days) was assessed. Antibacterial dental composites using QADM on an overlying mature cariogenic biofilm formed over seven days have not been studied to date. A longer-term in situ study would give insight into the in vivo antibacterial performance of this material in challenging conditions that mimic the clinical scenario of retentive proximal areas where the biofilm could not be removed in patients with a high risk of caries. Moreover, over a seven-day period, dysbiosis was present due to the proliferation or overgrowth of cariogenic bacteria in a low-pH econiche, and enamel was prone to demineralization.

In light of the evidence available to support quaternary ammonium monomers on initial oral biofilm, the present study for the first time evaluated the antibacterial performance of QADM in a relatively long-term study (beyond three days) by challenging the effectiveness of QADM-containing materials against mature oral biofilms formed in situ. Intact oral biofilms were grown under a cariogenic challenge in situ on composites within the oral cavity for 7 and 14 days. In addition to the determination of bacterial counts, the chronological changes in the biofilm were also visualized by live/dead staining, and the percentages were measured.

## 2. Results

**Figure 1:**
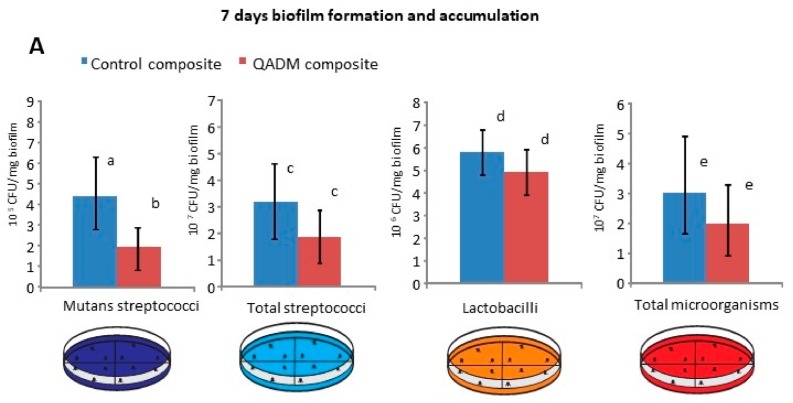

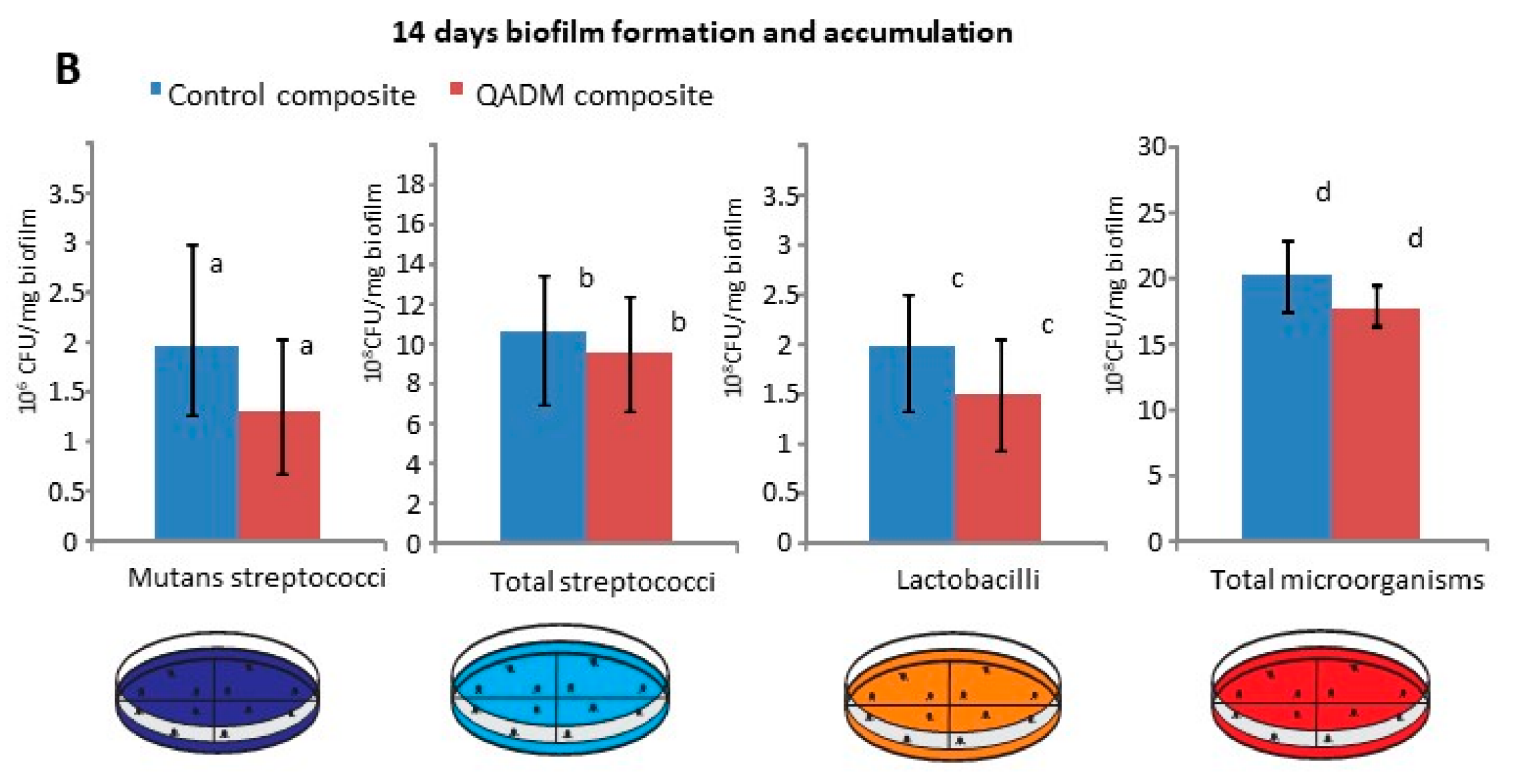
(**A**) Colony-forming unit (CFU) counts for the viability of mutans streptococci (MS), total streptococci (TS), lactobacilli, and total microorganisms (TM) present in the biofilms formed in situ after a 7-day and (**B**) 14-day period. Error bars represent the standard deviation of the mean, and data followed by different letters differed statistically (*p* < 0.05). The reduction in CFU counts from biofilms adherent to the QADM (bis(2-methacryloyloxyethyl) dimethylammonium bromide) composites was significantly different from the control for *Streptococcus mutans* after 7 days of growth. After 14 days, no further reduction was observed for *S. mutans*.

All 17 volunteers completed the study, and no protocol deviation was identified. Treatment compliance was satisfactory. The mean and standard deviation values of colony-forming unit (CFU) counts of biofilms collected at 7 and 14 days are plotted in Figure 1A,B. The QADM composite had a significant effect on the viability of *S. mutans* at the 7 days (*p* = 0.0303). This effect corresponded to a 43% reduction of both solutions compared with the control. The QADM composite’s effect on the viability of total streptococci, lactobacilli, and total microorganisms on the in situ biofilms was measured. However, no statistical significance was observed between the groups (*p* > 0.05). During the 14-day period, the microbiological composition of the biofilms formed on restoration was statistically similar for all evaluated conditions (*p* > 0.5).

**Figure 2:**
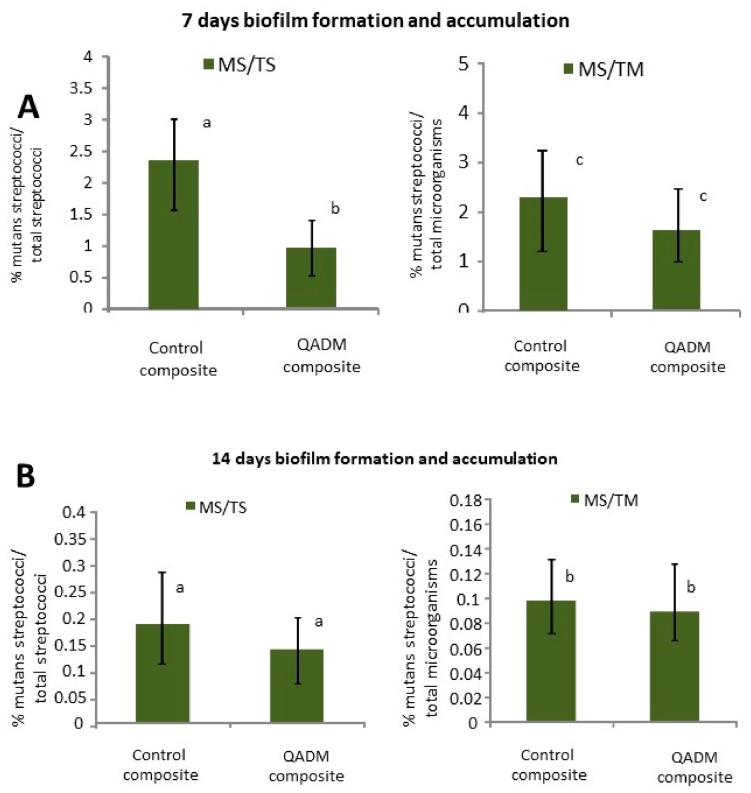
(**A**) The percentage of mutans streptococci related to total streptococci (MS/TS) and the percentage of mutans streptococci related to total micro-organisms (MS/TM) present in biofilms formed in situ after a 7-day and (**B**) 14-day period. The MS/TS was greatly reduced for biofilms adherent to the QADM composite in relation to the control at the 7-day period. Error bars represent SD, and data followed by different letters differed statistically (*p* < 0.05).

**Figure 3:**
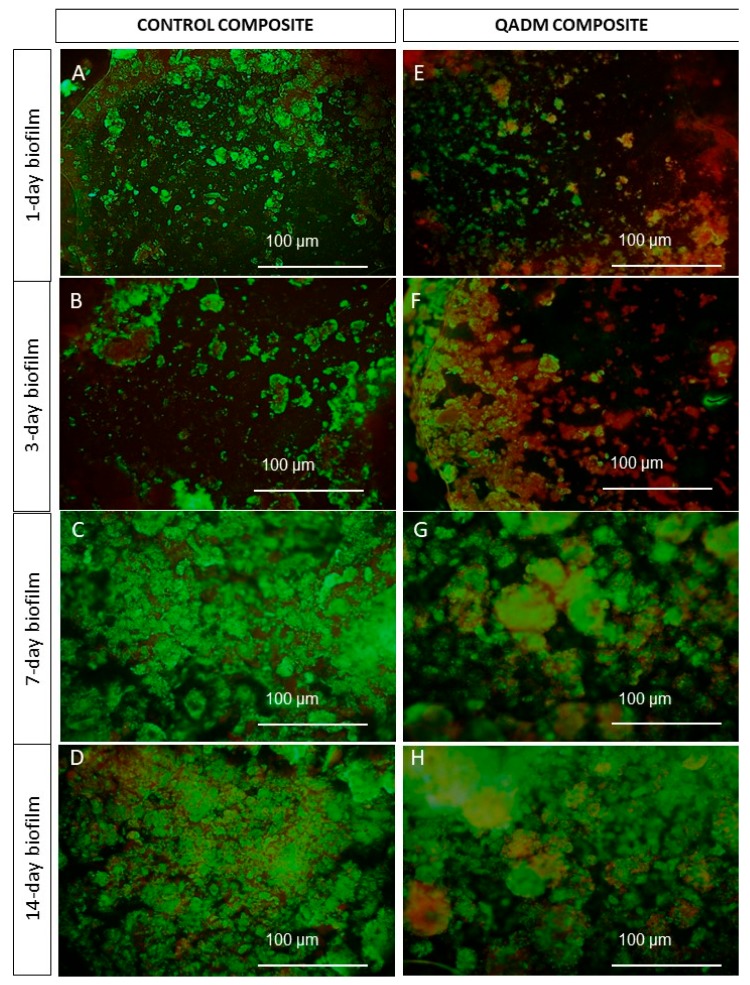
Live/dead staining images of biofilms grown on the QADM and control composites during the 1-, 3-, 7-, and 14-day periods. (**A**,**B**) Biofilms grown on the control composite on the 1st and 3rd days were primarily alive with continuous green staining. (**E**,**F**) Widespread cell-killing of bacteria was more pronounced on the 1- and 3-day biofilm accumulation on the QADM composite. On the 7th and 14th day, the overly mature biofilm structure was compact, with numerous layers presenting a mushroom-like configuration with channels in the outer layer: (**C**,**D**) for the control and (**G**,**H**) for the QADM.

Figure 2A shows the statistically significant difference between the tested groups (*p* = 0.0385) at the 7-day period, expressed by the variable percentage of mutans streptococci related to total streptococci (MS/TS). However, the percentage of mutans streptococci related to total micro-organisms was similar during the same period. These variables showed no difference during the 14-day period (Figure 3B).

Figure 3A–D,F–I shows live/dead staining images of biofilms grown on the QADM and control composites on the 1st, 3rd, 7th, and 14th days. Biofilms grown on the control composite on the 1st and 3rd days were primarily alive, which was indicated by continuous green staining (Figure 3A,B). Widespread bacterial cell killing was more pronounced on the 1st and 3rd day of biofilm accumulation on the QADM composite (Figure 3). At the 7th and 14th day, the overly mature biofilm structure was compact, with numerous layers showing a mushroom-like configuration with channels in the outer layer (Figure 3C,D for the control composites and Figure 3G,H for the QADM composites). Complete coverage of the composite surface was observed after seven days. No significant difference between the control and the QADM was observed.

**Figure 4:**
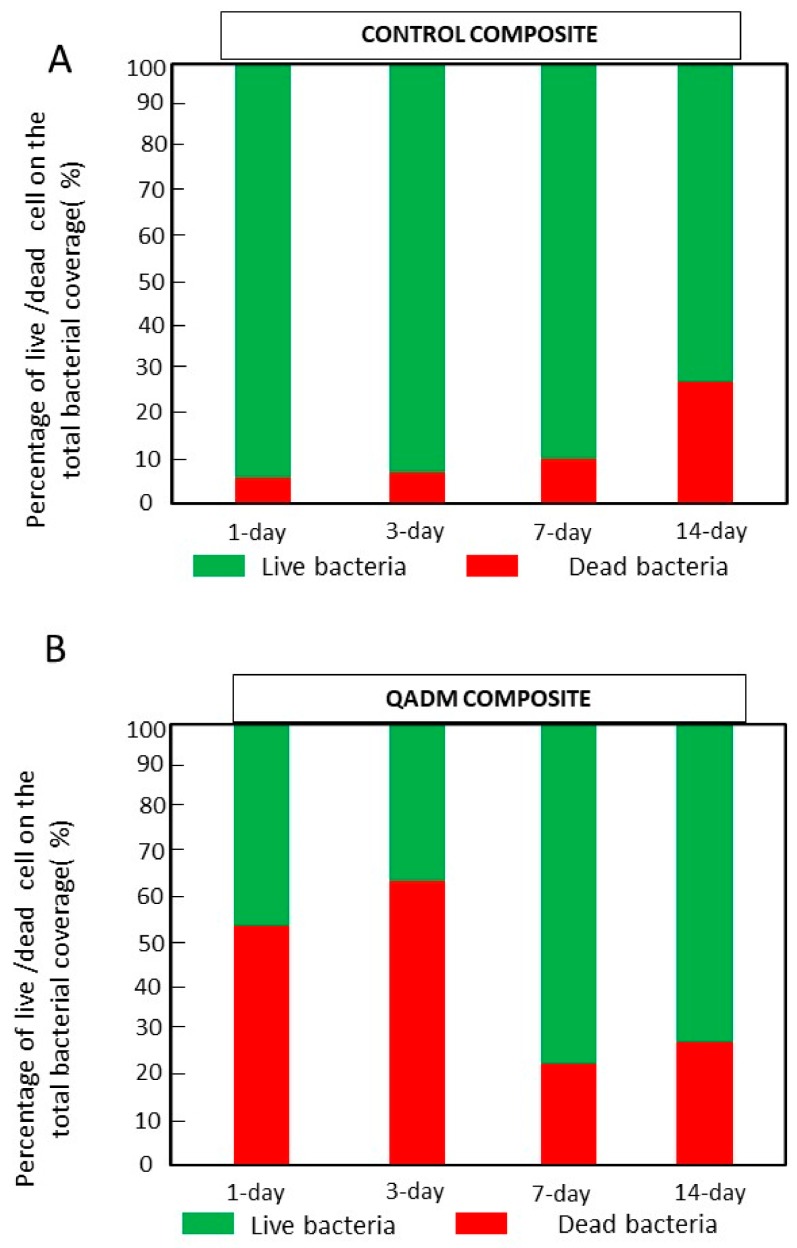
The percentage of live/dead bacterial cells found for the total biofilm coverage over the (**A**) control and (**B**) QADM composites. An increase in the dead percentage was observed for the biofilm grown over the QADM composite during the first and third day.

Figure 4 image analyses show that the living cells grown over control composites accounted for 93% ± 3% (±SD) and 93% ± 7% (±SD) of the total biofilm cells for the 1- and 3-day biofilms. Percentages of living cells (46% ± 8% for the first day and 37% ± 3% for the third day) were determined on the QADM-containing composites for the same time. They were inactive and dead.

## 3. Discussion

Bacterial attachment to dental composite surfaces and subsequent cariogenic biofilm formation is a complex process [26]. There is an interplay between biological factors (i.e., the bacterial ability to rapidly convert dietary sugars to acids lowers the pH and demineralizes the tooth structure), patient-related factors, and physicochemical factors such as surface topography, surface charge, and surface energy of the dental materials [27,28]. Typical treatment for biofilm-mediated recurrence of caries lesions around the composite restorations involves operative replacement of composites, which incurs additional healthcare costs and additional loss of tooth structure.

To avoid biofilms on dental materials, an attractive alternative and complementary method to dental caries management is the use of biomaterials that possess antibacterial surfaces [27]. The contact-active antibacterial material is effective in preventing biofilm formation by killing bacteria [29], reducing bacteria amounts in the surrounding microenvironment, and extending the material’s service life.

Antibiofilm effects of new dental materials with releasing fillers or ions were also investigated in the literature [30]. The incorporation of silver nanoparticles was among the bioactive compounds investigated that could provide antibacterial effects on oral bacteria. However, long-term release kinetics have challenged this approach’s use in dental materials [31,32].

As contact-killing agents, the quaternary ammonium monomers for dental applications have been intensely investigated in vitro in the past years, with a positive outcome overall. Investigations have shown immediate and robust antibacterial effects (more than 3 log reductions) against oral micro-organisms [21,23,25]. QADM di-functional monomers are effective in producing active surfaces with high densities of immobilized antimicrobial agents [33,34]. For the specific quaternary ammonium used in our study, the previous in vitro studies showed reductions of 68% and 79% in biofilm CFU counts and lactic acid production, respectively, on cured primers specimens [20].

For this in situ study, for the first time we challenged the antibacterial performance of a dual methacrylate group QADM incorporated into composites against overly mature oral biofilms formed inside the oral cavity for up to 14 days in an acidogenic biofilm structure capable of causing substantial mineral loss and deep lesions on the enamel surface. This approach has high clinical relevance since anti-caries therapies aimed at controlling the assembly of cariogenic biofilms should contribute to the prevention of the onset of early carious lesions, clinically known as white spots.

The data revealed that QADM compromised the *S. mutans* group’s biofilm accumulation during the 7-day period. The two most common species constantly linked to caries formation are *S. mutans* and *S. sobrinus* [35]. The cariogenic potential of *S. mutans* is accentuated when sucrose is available [36]. Sucrose-mediated biofilm formation creates spatial organizations expressed by a complex network of microcolonies, which modulate the development of compartmentalized acidic microenvironments across the 3D biofilm architecture [37]. Furthermore, within 3D biofilms, *S. mutans* display properties that are dramatically distinct from their planktonic counterparts, including much higher resistance to antibacterial approaches, which makes the biofilm much more difficult to kill than planktonic bacteria [35].

Although our research revealed the inhibitory effect of QADM on *S. mutans* biofilms, the exact mechanism of this inhibition is still unclear. The antibacterial efficacy may be related to the contact-killing mechanism of quaternization of the amino groups of QADM available on the bottom layer of the biofilm adjacent to the composite. The negatively charged counter-ions that stabilize the bacterial membrane were displaced by the positively charged cationic N^+^ sites in the chemical structure of the quaternary ammonium-based resin. Indeed, the live/dead images obtained at the initial period of biofilm formation showed the presence of a higher proportion of nonviable bacteria (the red-orange color areas). Previous studies have highlighted the similar viability of biofilms growing on resin-based materials containing quaternary ammonium monomers [35,36,38,39]. Beyth and co-workers have suggested an intracellularly mediated death program, in which the bacterial lysis promoted by the presence of quaternary ammonium on the resin surface functions as a stressful condition triggering programmed cell death in the bacteria further away in the biofilm [25,40].

No expressive microbial reduction results on CFU values or micro-organism proportions were observed for *S. mutans*. These results point out the challenge faced by anti-caries approaches against mature biofilms. The bacterial adhesion processes under in vivo and in vitro conditions differ considerably [33]. Bacteria in biofilms are far less sensitive to antibacterial agents because of the exopolymeric matrix, extracellular polysaccharides, specific gene expression, and metabolic activity, all factors that protect antibacterial therapies to reach target bacteria [41]. The live/dead images of biofilms show a well-developed dense and compact extracellular polymeric substance - EPS matrix and the presence of bacterial cell clusters or microcolonies (Figure 4C,D for the control composites and Figure 4G,H for the QADM composites). The relative alteration of the proportion of live/dead found in the 7–14-day images was related to the uneven spatial distribution of vital and dead microorganisms found in matured and thick dental biofilms, with decreased vitality toward the outer layers [41]. Tawakoli et al. [42] also supported the high variability of the live/dead distribution and the CFU counts as challenges found in in situ biofilm models. Recently, new investigations have started to emerge using nanoparticles to improve the penetration of therapeutic agents into the biofilm matrix of oral cariogenic biofilms [43].

Another aspect to consider is the spatial arrangement, charge density, and counter-anion of the quaternary ammonium monomers and their antibacterial activity [32]. Previous studies have designed antibacterial monomers containing an eight-carbon or longer chain, which has correlated with significant antibacterial activity in vitro [44]. Surface charge density has displayed antibacterial performance in vitro. These monomers need to translate their antibacterial performance from in vitro to a human in vivo [45,46,47,48]. Future studies are warranted, especially to investigate whether the charge density is a relevant factor in the antibacterial effect of these new quaternary ammonium monomers.

In summary, the findings in this paper demonstrate that QADM composites at 10% promote a substantial bacterial reduction of *S. mutans* biofilm. This was achieved in the initial days of contact and also reached a 7-day period, an interval where patients at high risk of caries would develop initial enamel carious lesions. However, dental caries result from interactions over time. An undisturbed cariogenic biofilm well-established on the composite surface over long periods is extremely difficult to eradicate. Its inhibition should not rely only on contact with an antibacterial surface. Removing or disturbing biofilm from all tooth and composite surfaces and reducing sugar intake within three days is expected to control carious lesions. Concomitant and multitargeting strategies are needed against mature long-term cariogenic biofilms.

## 4. Materials and Methods

### 4.1. Study Design and Participants

This study involved a prospective, randomized, single-blind, split-mouth in situ design conducted according to the code of ethics of the World Medical Association (Declaration of Helsinki) for experiments involving humans. The region’s ethical committee (protocol #1232012) also approved it. Seventeen healthy volunteers of both genders, aged from 21 to 36 years, accepted participation in this study, fulfilling the required criteria. Inclusion criteria were a normal salivary flow rate, good general and oral health with no active caries lesions or periodontal treatment needs, an ability to comply with the experiment protocol, no use of antibiotics during the three months before the study, and no use of fixed or removable orthodontic devices. Exclusion criteria were failing to use the device according to the established protocol and taking medication interfering with saliva flow rate or containing antimicrobial agents. The sample size was determined by a power analysis and was based on previous data [25]. Seventeen volunteers were recruited: 16 for the study and 1 volunteer to allow temporal visualization of the biofilm formation through live/dead staining. After being screened, the volunteers were verbally informed about the study aims and procedures, and received written information and the informed consent form. At the next appointment, maxillary alginate impressions were made for the fabrication of the palatal devices.

**Figure 5:**
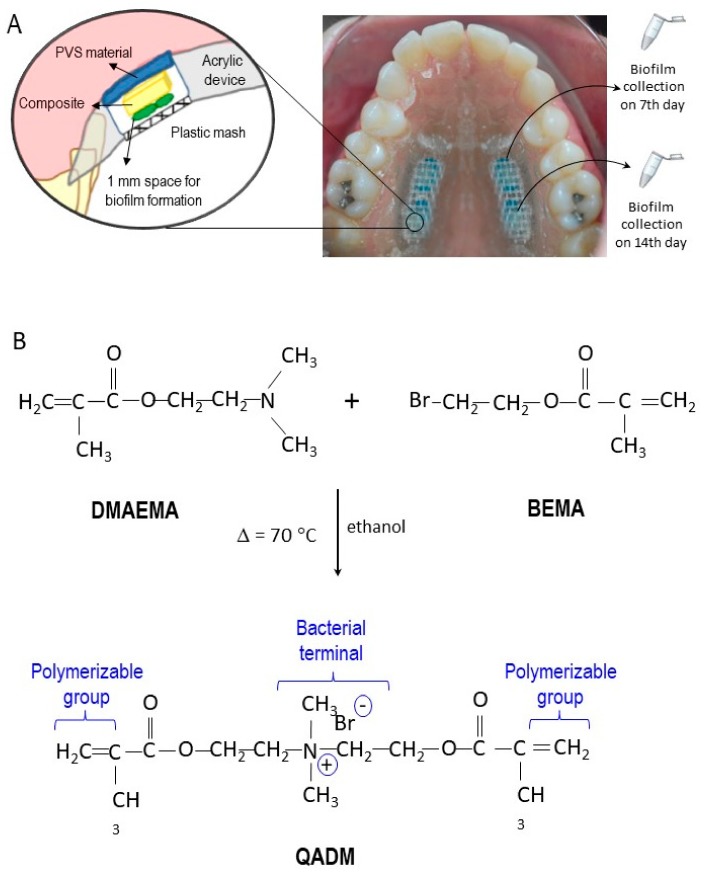
(**A**) In situ palatal devices used by 17 volunteers. Each device contained four slabs, two filled with the QADM composite on one side and two filled with the control composite on the other side. The slabs were held by polyvinyl siloxane (PVS) and covered with a plastic mash to avoid disturbance in the biofilm growth.The biofilm was collected from the surface of the specimens on the 7th and 14th days. The top-left is a magnified description showing details of biofilm formation over the composite specimens inside the device. (**B**) The synthesis route of the bis(2-methacryloyloxyethyl) dimethylammonium bromide monomer via a Menshutkin reaction and the details for dual-polymerizable groups and the bacterial terminal. BEMA: 2-bromoethyl methacrylate; DMAEMA: 2-(*N,N*-dimethylamino)ethyl methacrylate.

During the experimental period, each volunteer used a removable acrylic custom-made palatal device containing the tested materials, as shown in Figure 5A. Seven days before the experiment began (i.e., the washout period) and during the whole experiment, the volunteers were asked to use a standard toothbrush and nonfluoridated paste. Each acrylic palatal device enclosed four composite specimens (5 × 5 × 2 mm^3^): two specimens for the control composites and two specimens for the QADM composites. To promote plaque accumulation and to protect it from disarrangement, recessions were created by placing the surface of the composite specimens about 1 mm below the covered plastic mesh (as seen in the details of Figure 1A) [45].

To divert any possible carry-across effect, the sequence in which the experiment units were assigned in the palatal device took into consideration that antibacterial dental materials should be placed on one side of the palatal appliance and, consequently, that control materials should be placed on the opposite side (Figure 5A). The split-mouth experimental design was a practical approach for testing the effects of various agents on the composition of dental plaque [44]. Within each side of the palatal device, the positions of the specimens were randomly determined according to a computer-generated randomization list [45]. The outcome variables evaluated were colony-forming unit counts for total microorganisms, total streptococci, mutans streptococci, and lactobacilli on the specimens.

### 4.2. Specimen Preparation

The light-curable composite was made by blending a monomer resin consisting of BisGMA (bisphenol-glycidyl dimethacrylate) and TEGDMA (triethylene glycol dimethacrylate) at a 1:1 ratio (all by mass) with 0.2% camphorquinone and 0.8% ethyl 4-*N,N*-dimethylaminobenzoate. As reinforcement co-fillers, barium-boroaluminosilicate glass particles with a median diameter of 1.4 µm (Caulk/Dentsply, Milford, DE, USA) were silanized with 4% 3-methacryloxypropyltrimethoxysilane and 2% *n*-propylamine [21]. The fillers were mixed with the resin at a total filler mass fraction of 60% to form a cohesive paste.

The synthesis of bis(2-methacryloyloxyethyl) dimethylammonium bromide via a modified Menshutkin reaction was previously described [18], and it is summarized in Figure 1B. Briefly, 10 mmol of 2-(*N,N*-dimethylamino)ethyl methacrylate (DMAEMA; Sigma-Aldrich, St. Louis, MO, USA) and 10 mmol of 2-bromoethyl methacrylate (BEMA; Monomer-Polymer Labs, Trevose, PA, USA) were combined with 3 g of ethanol in a closed vial. After stirring at 60 °C for 24 h so the reaction could complete, the solvent was removed via evaporation, using a vacuum. This process yielded QADM as a clear and viscous liquid. QADM was mixed with the BisGMA–TEGDMA resin at a QADM mass fraction of 10%. A preliminary study showed that this mass fraction yielded strong antibacterial properties without compromising the resin’s mechanical properties [21].

Thirty-six light-curable composite specimens were fabricated for the experimental composition (QADM at 10 wt % each), and a further 36 specimens without antimicrobial monomer served as a control. The composite was inserted and light activated for 20 s using a light-emitting diode (Radii-cal, SDI Limited Victoria, Australia; standard curing mode, irradiance output provided of 689 mW/cm^2^). The specimens were mounted in standardized sample chambers inside the device, with an anterior–posterior position, using body impression material (Aquasil Ultra, Dentsply DeTray GmbH, Konstanz, Germany), as demonstrated in Figure 1A.

### 4.3. Clinical Phase

Audiovisual orientation and written instructions of the in situ protocol were given to the volunteers to assure their adhesion and avoid protocol deviation during the study. To provide a cariogenic challenge during the clinical phase, the application of a 20% sucrose solution extra-orally on the restored specimens was performed by the volunteers eight times per day at predetermined times. According to previous studies, the sucrose was gently dried after 5 min, and the device was reinserted into the mouth [42,44]. No restriction was made about the volunteers’ diet, but they were instructed to avoid F-rich food containing bioavailable F, such as black tea. They did, however, drink fluoridated water (about 0.7 ppm fluoride).

### 4.4. Microbiological and Biochemical Analysis

On the 7th and 14th examination days, the subjects refrained from eating, drinking, and tooth cleaning 12 h after the last application of the sucrose solution and dentifrice before presenting at the clinic (46-48). On the 7th day, the device was removed from the mouth, and the biofilm and one enamel slab from each side were respectively carefully removed and collected (Figure 5B). Then, the device with the remaining specimens was reinserted into the mouth. On the 14th day, a similar process was performed to collect the two residual biofilms from the specimens. After the collection, the biofilm was processed for analysis. First, it was weighed (±1 mg) in preweighed microcentrifuge tubes and agitated during a 2 min period in a Disrupter Genie Cell Disruptor (Precision Solutions, Rice Lake, WI, USA). A 50 µL aliquot of the sonicated suspension was diluted in 0.9% NaCl, and serial decimal dilutions were inoculated in triplicate using the drop-counting technique in the following culture media: (1) in mitis salivarius agar containing 20% sucrose to determine total streptococci (TS), and in mitis salivarius agar plus 0.2 bacitracin/mL to determine mutans streptococci (MS); (2) in Rogosa agar supplemented with 0.13% glacial acetic acid to assess the number of CFU of lactobacilli (LB); and (3) in brain–heart infusion enhanced with 5% sterile defibrinated sheep blood agar plates to determine total micro-organisms (TM). The plates were incubated in 10% CO_2_ at 37 °C for 48 h. The CFU were counted, and the results were expressed as CFU/mg biofilm wet weight, the percentage of MS in relation to TM, and the percentage of MS in relation to TS.

### 4.5. Live/Dead Assays

To visualize the micro-organisms during the initial phase of formation as well as during the experimental periods, one volunteer used a palatal device containing eight composite specimens: four specimens for the control composite and four specimens for the QADM composite. One specimen from each group was removed during the 1st, 3rd, 7th, and 14th days. The specimens were immediately washed with phosphate-buffered saline (PBS) and stained using the LIVE/DEAD BacLight Bacterial Viability Kit (Molecular Probes, USA) to qualify bacterial cell viability. This assay employs two nucleic acid stains: the green-fluorescent SYTO 9 stain and the red-fluorescent propidium iodide stain [39]. These stains differ in their ability to penetrate healthy bacterial cells. When used alone, the SYTO 9 stain labels both live and dead bacteria.

In contrast, propidium iodide penetrates only bacteria with damaged membranes, reducing SYTO 9 fluorescence when both dyes are present. Thus, live bacteria with intact membranes fluoresce green, while dead bacteria with damaged membranes fluoresce red. A volume of 100 μL of the previously described fluorescence dyes was pipetted onto the specimens and incubated in a dark chamber for 15 min. The biofilms grown over the specimens were then examined using an epifluorescence microscope (TE2000-U, Nikon, Melville, NY, USA) at a magnification of 100×. Images (*n* = 4) were acquired and analyzed (NIS Elements software, Nikon Instruments Inc, Melville, NY, USA) for the quantification of live (green fluorescence) and dead (red fluorescence) bacteria.

### 4.6. Statistical Analysis

The assumptions of equality of variances and normal distribution of errors were checked for all the response variables tested, and those that did not satisfy these assumptions were transformed using the Box–Cox power transformation [42]. To determine the differences between test and control values in the in situ experiment, the viable bacteria counts, percent MS/TS, and percent MS/TM were submitted to a two-sample independent Student’s *t*-test. The significance level was set at α = 0.05. The statistical appraisal was computed with SPSS for Windows XP 17.0 (SPSS Inc., Chicago, IL, USA).

## 5. Conclusions

The results of the present in situ study provide a more realistic perspective on the value of integrating bioactive restorative materials with traditional caries management approaches into clinical practice. Contact-killing strategies via dental materials aiming at preventing or at least reducing high numbers of cariogenic bacteria seem to be a promising approach for helping patients at high risk of recurrence of dental caries around composites.
